# How do Vaccinators Experience the Pandemic? Lifestyle Behaviors in a Sample of Italian Public Health Workers during the COVID-19 Era

**DOI:** 10.3390/vaccines10020247

**Published:** 2022-02-06

**Authors:** Francesca Gallé, Alessia Quaranta, Christian Napoli, Giusy Diella, Osvalda De Giglio, Giuseppina Caggiano, Marco Di Muzio, Pasquale Stefanizzi, Giovanni Battista Orsi, Giorgio Liguori, Maria Teresa Montagna

**Affiliations:** 1 Department of Movement Sciences and Wellbeing, University of Naples Parthenope, 80133 Naples, Italy; francesca.galle@uniparthenope.it (F.G.); giorgio.liguori@uniparthenope.it (G.L.); 2 Department of Biomedical Science and Human Oncology, University of Bari Aldo Moro, 70124 Bari, Italy; alessia.quaranta@uniba.it (A.Q.); giusy.diella@uniba.it (G.D.); osvalda.degiglio@uniba.it (O.D.G.); giuseppina.caggiano@uniba.it (G.C.); pasquale.stefanizzi@uniba.it (P.S.); mariateresa.montagna@uniba.it (M.T.M.); 3 Department of Medical Surgical Sciences and Translational Medicine, Sapienza University of Rome, 00189 Rome, Italy; 4 Department of Clinical and Molecular Medicine, Sapienza University of Rome, 00185 Rome, Italy; marco.dimuzio@uniroma1.it; 5 Department of Public Health and Infectious Diseases, Sapienza University of Rome, 00185 Rome, Italy; giovanni.orsi@uniroma1.it

**Keywords:** COVID-19 pandemic, public health worker, lifestyle

## Abstract

Public health workers (PHWs) have experienced substantial workload changes because of their role in managing measures to limit the spread of COVID-19. The study’s aim was to assess lifestyle changes in Italian PHWs during the pandemic. PHWs attending an annual meeting completed an anonymous questionnaire assessing their sociodemographic and behavioral characteristics and lifestyle changes during the pandemic. A total of 1000 questionnaires were completed. Most participants (63.5% women, mean age 40 ± 13.1 years) were of normal weight (61.5%), non-smokers (81.9%), had a total screen time of ≥5 h/day (83.1%), and slept at least 6 h/night (88.7%). Approximately one-third consumed sweet foods every day (30%) and did not engage in physical activity (34.6%). Current sweet food consumption, physical activity, and sleep were associated with changes in these behaviors in the last 2 years (Tau-b = 0.155; Tau-b = −0.175; Tau-b = −0.276, respectively, *p* < 0.001). An increase in remote working was associated with worse sleep (odds ratio (OR) 2.065, 95% confidence interval (CI) 1.482–2.877) and diet (OR 1.982, 95% CI 1.385–2.838), and increased tablet/PC use (OR 3.314, 95% CI 2.358–4.656). Health promotion measures are needed to support the adoption of healthy lifestyles in this population during the current pandemic.

## 1. Introduction

The severe acute respiratory disease caused by the novel coronavirus SARS-CoV-2 (COVID-19) is a serious public health problem worldwide and has posed an unprecedented health and socioeconomic burden [1,2]. The virus was first reported in Italy in early 2020 and spread rapidly across the whole country in consecutive waves, causing thousands of hospitalizations and deaths [3,4].

In response to the epidemiological situation and drawing on previous experience of infectious disease control, the Italian government adopted a series of control measures to restrict the spread of the virus, such as restriction of movements, obligatory use of facial masks, contact tracing for early warning, and specific methods of environmental control measures, that were applied beside the routine microbiological checks [5,6,7,8].

Inevitably, these measures had substantial effects on citizens’ lifestyle, health, and psychophysical well-being. Several studies have been conducted in Italy to assess behavioral changes during lockdowns and throughout the ongoing COVID-19 pandemic in the general population and in specific populations such as healthcare workers (HCWs) [9,10,11]. With few exceptions, these studies report increasing psychological distress and the adoption of unhealthy behaviors, especially related to diet and physical activity (PA).

In particular for HCWs, several studies have showed how severely well-being has been affected during the pandemic in Italy. It was demonstrated that HCWs living in the most affected regions had a prevalence of psychological distress higher than their colleagues from the rest of the country; moreover, significant differences related to life changes were associated with the lockdown [12]. Another study revealed that one year after the beginning of COVID-19 emergency, Italian nurses were at the greatest risk of anxiety and depression, whereas residents were at the greatest risk of burnout, and working in intensive care units was associated with an increased risk of developing severe emotional exhaustion and a cynical attitude towards work [13].

A repeated cross-sectional study shows that one year after the baseline evaluation, Italian HCWs reported an increased workload, isolation at work and in their social life, and a lack of time for physical activity and meditation [14]. The level of these working-related unhealthy effects was higher in HCWs who directly managed the COVID-19 emergency compared to those who were not directly involved [15].

In this context, public health workers (PHWs) are health care workers dealing with preventive medicine and health promotion. With regard to the pandemic, apart from the managing role of evaluating and providing the best setting-specific procedures for disease prevention, PHWs were responsible for some crucial and direct control measures such as contact tracing and related screening by molecular testing targeted to both the general population (in *drive-thru* testing centers) and home quarantined contacts. Moreover, PHWs were responsible for the immunization campaigns when the first vaccines became available (vaccinating dozens of millions of Italians). Therefore, PHWs were not involved in the “clinical” management of severe cases of diseases, but they have had contact with COVID-19 patients and filled a role of high responsibility in the disease control. To our knowledge, while some studies have been published regarding the pandemic’s effects on HCWs, no studies have analyzed health-related behaviors adopted by PHWs during the pandemic.

The aim of this study was to explore lifestyle changes that have occurred since the beginning of the COVID-19 pandemic in Italian PHWs joining the Italian Society of Hygiene, Preventive Medicine, and Public Health (SItI) and to investigate associations between the self-reported changes and participants’ sociodemographic and behavioral characteristics. A second phase of the study will be conducted in November 2022 to test long-term self-reported consequences of the pandemic.

## 2. Materials and Methods

### 2.1. Participants and Setting

SItI represents PHWs working in both public national health system and in research institutions such as universities and public health institutions. In November 2021, the society’s annual meeting was held in Lecce, southern Italy. Meeting attendees were asked to complete an anonymous questionnaire. Participation was voluntary and attendees were informed that the completion of the questionnaire implied informed consent for data collection and treatment.

We considered as the target population the total number of PHWs joining the SItI (2240 PHWs). Based on the reference population and assuming a 5% margin of error and a 95% confidence level, the minimum sample size was estimated at 328 PHWs.

This study was performed in accordance with the World Medical Association’s Declaration of Helsinki. The study was approved by the scientific institutional review board of the Italian Inter University Research Centre “Population, environment and health” (approval number: 2810_2021).

### 2.2. Questionnaire

The questionnaire design was based on tools used in previous studies [16,17,18] and modified to fit the target population. The questionnaire was reviewed by a panel of experts comprising one epidemiologist, one public health professional, one psychologist, one expert in nutrition and movement sciences.

Moreover, the intelligibility of the questions was evaluated by asking a small separate sample to assign a 7 point score from 7 (very meaningful) to 1 (not meaningful at all) to each question. To this purpose, the original questionnaire, reporting the standard questions (SQ) was revised: 10 adjunct questions (AQs), reporting both semantic and grammatical errors, were added to the standard questions. The mean score for each SQ was >6 and for each AQ was <2. Therefore, the content of the questionnaire was considered clear to readers.

A preliminary pilot study involving 48 people was carried out to test the questionnaire’s validity (data not published). Three sections were developed:

#### 2.2.1. Personal Information

The first section recorded sociodemographic information such as gender, age, work institution (research/healthcare service institution), and living conditions (alone/with parents/with friends or colleagues/with partner/with parents and underage children/with parents and adult children). Participants were also asked to self-report their weight and height to enable calculation of body mass index (BMI) and related weight status (underweight/normal/weight/overweight/obese) according to the World Health Organization classification [19].

#### 2.2.2. Self-Reported Current Behaviors

The second questionnaire section comprised questions about current behaviors. Participants were asked to report if they smoked (no/yes); for how many days/weeks they ate sweet foods; if they engaged in PA (no/walking or cycling for commuting/walking or cycling for leisure/doing exercise or sport outdoors/doing exercise or sport in indoor facilities/doing exercise or sport at home); and how much they watched TV, used a smartphone, used a tablet/PC (<1/1/2/3/4/≥5 h/day), and slept (≤5/6/7/8/≥9 h/night) at the time of the investigation. Cronbach’s alpha (internal consistency coefficient) was used to test the reliability of both the pilot and final study [20]. The alpha values for this section showed a good level of reliability (0.74 and 0.70, for the pilot and the final study respectively) [21].

#### 2.2.3. Self-Reported Current Behaviors Changes during the Pandemic

The third section of the questionnaire assessed lifestyle changes during the pandemic. Participants were asked to report changes in work activity (no change/more remote work/more onsite work); smoking habits (stopped/decreased/no change/started/increased); dietary habits (improved/no change/worsened); sweet food consumption (decreased/no change/started/increased); body weight (decreased/no change/increased); PA (started/increased/no change/decreased); time spent watching TV, using a smartphone, using a tablet/PC (decreased/no change/increased); and sleep time (increased/no change/decreased). In addition, for this section, the Cronbach’s alpha values showed a good level of reliability (0.86 and 0.82 for the pilot and the final study respectively) [21].

In addition, participants were asked a question about their COVID-19 vaccination status (“How many doses of COVID-19 vaccine have you received so far?”).

### 2.3. Statistical Analyses

Descriptive analysis was performed on participant sociodemographic, anthropometric, and behavioral characteristics. Continuous variables were expressed as the mean value ± standard deviation. Categorical variables and responses were reported as the number and percentage of respondents. Gender comparisons were performed using the chi-square test. Kendall’s correlation analysis was used to identify possible relationships between sociodemographic, anthropometric, and behavioral characteristics and vaccination status. To this aim, gender was categorized as female = 0 and male = 1; age as <median value = 0 and ≥median value = 1; research institution = 0, healthcare institution = 1; living alone = 0, with parents = 1, with friends/colleagues = 2, with partner = 3, with parents and underage children = 4, with parents and adult children = 5; BMI as underweight = 0, normal weight = 1, overweight = 2, and obese = 3; non-smoker = 0, smoker = 1; and PA non-engagement = 0, walking/cycling for commuting = 1, walking/cycling for leisure = 2, exercise/sport outdoors = 3, exercise/sport in indoor facilities = 4, exercise/sport at home = 5. Days/week of sweet food consumption and hours/day of watching TV, using a smartphone, using a tablet/PC, and sleeping hours/night were categorized according to their corresponding frequencies. Lifestyle changes were categorized as follows: no changes in work activity = 0, more remote work = 1, more onsite work = 2; smoking stopped = 0, decreased = 1, no change = 2, started = 3, increased = 4; dietary habits improved = 0, no change = 1, worsened = 2; sweet food consumption decreased = 0, no change = 1, started = 2, increased = 3; body weight and time spent watching TV, using a smartphone, and using a tablet/PC decreased = 0, no change = 1, increased = 2; PA engagement started = 0, increased = 1, no change = 2, decreased = 3; sleep time decreased = 0, no change = 1, increased = 2.

A multivariate logistic regression analysis was performed to identify variables associated with lifestyle worsening. Changes in smoking; sweet food consumption; diet; weight; PA; time spent watching TV, using a smartphone, and using a tablet/PC; and sleep time were considered dependent variables and codified as improved/no change = 0, worsened = 1. Gender, age, work institution, living conditions, and changes in work activity were considered as independent variables.

A *p*-value of 0.05 was assumed to indicate significance. Analyses were performed using the software IBM SPSS version 27 for Windows (IBM Corp., Armonk, NY, USA).

## 3. Results

Of 1463 PHWs attending the society meeting, 1024 completed the questionnaire. After data cleaning, 1000 questionnaire were deemed complete and correctly filled in and were used for analysis. Table 1 shows the main characteristics of the sample.

The sample comprised mainly women, individuals working in research institutions, and individuals living alone or with parents. The mean age was 40 years (approximate range: 20–80 years).

Table 2 shows participant responses about current lifestyle behaviors and characteristics, and the number of COVID-19 vaccine doses received at the time of the investigation.

Regarding lifestyle characteristics and behaviors, most participants were of normal weight and non-smokers. However, approximately one-third of participants reported the consumption of sweet foods every day (30%) and did not engage in PA (34.6%). A high percentage of participants had a total screen time reaching/exceeding 5 h/day (83.1%) and slept at least 6 h/night (88.7%). Most PHWs had received at least two COVID-19 vaccine doses at the time of the investigation. Significant gender differences were found for BMI, smoking, PA, time spent using tablets/PCs, and vaccine dose. Regarding these aspects, women showed more healthy behaviors than men, with the exception of PA and using tablets/PCs. A higher proportion of male respondents reported a third dose of COVID-19 vaccine.

Table 3 highlights changes in participants’ lifestyles during the pandemic.

Most participants reported changes (including increases and decreases) in work activity, PA (almost 39% reported decreased PA), and time spent using tablets/PCs (increased in more than 56% of the sample). Regarding work, an equal percentage of participants (26.2%) declared increases in remote and onsite work. The proportions of participants who reported no change versus a decrease in PA were similar. The gender comparison showed that women experienced higher changes than men in dietary habits, sweet food consumption, time spent watching TV and using smartphone, and in sleep time.

The correlation analyses showed that current smoking was negatively related to age (tau-b = −0.090, *p* = 0.004), living conditions (tau-b = −0.079, *p* = 0.005), current PA (tau-b = −0.070, *p* = 0.014), and sleep time (tau-b = −0.066, *p* = 0.023), and positively related to gender (tau-b = 0.070, *p* = 0.027), current TV watching (tau-b = 0.064, *p* = 0.027), smartphone use (tau-b = 0.148, *p* < 0.001), and total screen time (tau-b = 0.074, *p* = 0.006). Current smoking was also related to changes during the pandemic in work activity (tau-b = 0.082, *p* = 0.006) and weight (tau-b = 0.071, *p* = 0.018).

Current consumption of sweet foods was negatively related to current PA (tau-b = −0.094, *p* < 0.001) and positively related to current TV watching (tau-b = 0.081, *p* = 0.002), smartphone use (tau-b = 0.065, *p* = 0.009). This variable was also related to pandemic changes in weight (tau-b = 0.082, *p* = 0.002), dietary habits (tau-b = 0.147, *p* < 0.001), sweet food consumption (tau-b = 0.155, *p* < 0.001), and PA (tau-b = 0.058, *p* = 0.028).

Current PA was negatively related to age (tau-b = −0.086, *p* = 0.003), BMI (tau-b = −0.190, *p* < 0.001), living conditions (tau-b = −0.074, *p* = 0.003), and current sweet food consumption (tau-b = −0.094, *p* < 0.001). Current PA was also related to pandemic changes in smoking (tau-b = −0.150, *p* = 0.019), diet (tau-b = −0.197, *p* < 0.001), sweet food consumption (tau-b = −0.144, *p* < 0.001), and PA (tau-b = −0.175, *p* < 0.001).

Current total screen time was positively related to current consumption of sweet foods (tau-b = 0.080, *p* = 0.001) and smoking (tau-b = 0.074, *p* = 0.006) and to pandemic changes in work activity (tau-b = 0.091, *p* < 0.001), PA (tau-b = 0.080, *p* = 0.002), dietary habits (tau-b = 0.105, *p* < 0.001), sweet food consumption (tau-b = 0.084, *p* = 0.001), and sleep time (tau-b = 0.063, *p* = 0.017).

Current sleep time was negatively related to age (tau-b = −0.172, *p* < 0.001), BMI (tau-b = −0.071, *p* = 0.011), living conditions (tau-b = −0.073, *p* = 0.004), and current smoking (tau-b = −0.066, *p* = 0.023). Current sleep time was also related to pandemic changes in weight (tau-b = −0.055, *p* = 0.044), dietary habits (tau-b = −0.074, *p* = 0.008), and sleep (tau-b = −0.276, *p* < 0.001), and positively related to current time spent watching TV (tau-b = 0.093, *p* = 0.001).

Table 4 shows the results of the logistic regression analysis.

These results show that increased smoking was positively associated with living with parents and underage children and negatively associated with an increase in remote working during the pandemic. Worsening diet, sweet food consumption, body weight, and PA were more common in younger participants and less frequent in those who worked in research institutions. Dietary and sweet food consumption changes were also associated with living with a partner and adult children. Remote working was positively associated with diet worsening. Female gender was positively related to sweet food consumption. Living with friends/colleagues was negatively related to weight increase and positively related to PA decrease during the pandemic. Regarding screen time, increased TV watching, and smartphone use were associated with an increase in onsite working, and tablet/PC use was related to living with a partner and underage children and to increase in both onsite and remote working. Sleep worsening was negatively related to working in a research institution and with living with a partner and adult children and positively associated with an increase in remote working.

## 4. Discussion

This study identified the self-reported lifestyle changes experienced during the COVID-19 pandemic by Italian PHWs, professionals responsible for health promotion and disease prevention policies. During the last 2 years, these frontline workers have implemented policies to control the spread of COVID-19, particularly policies related to the national COVID-19 immunization campaign.

Several studies have been conducted on the effect of the pandemic on HCWs, focusing on contagion and deaths, adherence to control measures, and psychological effects; however, few studies have investigated the effect of the pandemic on lifestyle [11,22]. To the best of our knowledge, none of these studies have specifically investigated PHWs.

The main findings of this study show that the current health-related habits and characteristics of this population differ in some respects from those of the general Italian population. In fact, although most participants did not smoke and were of normal weight at the time of the investigation, a substantial proportion were physically inactive and reported daily sweet food consumption.

A 2020 Italian Institute of Statistics report showed that of the general Italian population of the same age category as our sample, approximately 18.2% were smokers, approximately 47% were overweight/obese, and approximately 31.6% did not engage in PA [23]. Our sample showed the same percentage of smokers as in the general Italian population, a lower proportion of overweight/obese participants, and a higher proportion of inactive participants.

Regarding gender differences, women had healthier lifestyles than men, with the exception of PA and time spent using tablets/PCs. This finding is inconsistent with a study of Italian undergraduate students, which showed that being female was associated with the achievement of recommended PA levels even during lockdown [18]. This difference could be explained by factors not investigated in the present study, such as psychological conditions more frequently observed in female HCWs than in the general population [24].

The aim of the present study was to assess changes in PHW lifestyle during the pandemic. The observed changes in weight, diet, PA, and sleep were more positive than those found in a previous study of Italian older adults [25]. In another study performed among Italian not-HCWs, a lower percentage of participants reported a decrease in PA [18]. Although that study investigated only individuals of a narrow age range and was restricted to the lockdown period, comparison with the present findings confirms the differences between PHWs and the general Italian population.

Our findings on changes in PA, screen time, and sleep are consistent with the results of a previous Italian study that found substantial differences between HCWs and the general population [11]. However, a comparison of previous results for HCWs and the present data show differences between HCWs and PHWs.

Regarding smoking, more than 15% of our sample started, maintained, or increased smoking, higher than the 4.4% of smokers reported in a study of HCWs [22]. A web-based survey study found that during the lockdown, individuals marginally consumed more cigarettes (7.4%) compared with before the lockdown; however, participants to that study were part of an unselected sample and, therefore, not restricted to HCWs [26]. This result is important because smoking is one of the factors associated with lower antibody titers following COVID-19 vaccination [27]. In fact, smoking is associated with reduced immune system function, which is related to the development of autoimmune disease and reduced response to infection [28].

Regarding dietary habits, 75% of our sample showed either no change or adopted a healthier diet. This percentage was lower than that found in other studies, which have reported levels of healthier eating up to 96.8% in HCWs [22]. This finding is of note considering the importance of a good diet for health and particularly for protection against COVID-19. In a study of HCWs in six European countries, plant-based or pescatarian diets were associated with lower likelihood of developing moderate-to-severe COVID-19, suggesting that a balanced diet protects against severe COVID-19 [29]. This is probably because a healthy diet supports the immune system (e.g., by increasing antibody production and lymphocyte proliferation) and reduces oxidative stress [30]. Both specific micronutrient deficiencies and generalized malnutrition are associated with host immune dysfunction [31]. Moreover, the COVID-19 virus may be affected by host nutritional deficiency; it may be more virulent if it replicates in a nutritionally deficient host [29]. Moreover, there is preliminary evidence that some nutrients may modulate stress resilience in humans [32], which could help in planning healthy meals for HCWs [33].

We found that 61.1% of our sample experienced unchanged or increased PA levels during the pandemic. This percentage is lower than that found in other studies, which report PA levels of up to 68.2% in HCWs [22]. Almost 40% of our sample reported a reduction in PA. This is consistent with a study conducted in Singapore that demonstrated pandemic-related PA reduction [24]. PA reduction is a substantial risk factor for mild stress and moderate-to-severe depression [24]. A study by Woods et al. demonstrated that HCWs may have increased susceptibility to COVID-19 owing to weakened antiviral defenses from the prolonged disruption of PA [34].

Many HCWs lack access to PA because of an increase in work and lockdown restrictions. However, indoor exercise is recommended in these situations, following the World Health Organization 2020 Guidelines on Physical Activity and Sedentary Behaviour [24,35]. Interventions, such as sprint interval training to increase cardiorespiratory fitness and in-hospital promotion of PA (e.g., by making bicycles available in open spaces), can improve PA in HCWs [24,35].

Our data on the number of that received doses of the COVID-19 vaccine are heartening: most participants had received at least two doses. Only five participants had not been vaccinated at all. These individuals may have had vaccine contraindications and probably accessed by using negative swab test certification. These data are interesting because PHWs are one of the most important sources of vaccination information for the general public [36]. In particular, PHWs are engaged to promote immunization in different healthcare settings and to develop effective communication toolkits and educational materials about vaccination [36].

We found several significant correlations among PHW lifestyle characteristics. In particular, the different aspects of screen time were associated with smoking and sweet food consumption. Other studies have reported a clustering of unhealthy behaviors during the pandemic and have identified associations between such behaviors and mental health indicators [37,38].

The correlation analysis also suggested a possible association between changes during the pandemic and current behaviors. In particular, current sweet food consumption, PA, and sleep were associated with changes in the same behaviors in the last 2 years. These data agree with recent study findings [39].

The regression analysis confirmed some of these relationships and emphasized the effects of changes in work activity on health-related behaviors, as reported in previous studies [40]. In particular, a positive association was found between an increase in remote working and worsening diet and sleep, and with increased time spent on tablets/PCs.

These findings provide new perspectives on the effects of work changes on Italian PHWs lifestyle and health.

There are several study limitations. The survey was performed using a non-standard questionnaire. Although the questionnaire was structured on the basis of the tools used in previous studies and validated in terms of intelligibility and reliability, this represents the first limitation of the study. Secondly, the cross-sectional design of the study limits its validity in assessing temporal relationship between exposure and outcome. The questionnaire was administrated once, and self-report data were used, which make it prone to recall, reporting, and desirability biases; therefore, some of the effects may have been overestimated or underestimated. Anyway, a second evaluation is foreseen during the next SItI national congress that will be held in Padua in November 2022, as part of a repeated two-point cross-sectional survey. This methodology has been already used in previous studies, enabling to follow variations over time in the response of HCWs to the pressure posed by the COVID-19 pandemic [13,14,15]. Third, due to the reference population considered, the findings cannot be extended to the whole population of HCWs in Italy. Furthermore, in our study, being vaccinated and participating to a congress held in person in an epidemiological situation that at the time of the study was characterized by a low number of infections after the third wave end may have influenced respondents’ believing about the pandemic and answers about their own behaviors. For the same reasons we did not evaluated the number of PHWs experiencing the SARS-CoV-2 infection and the related health consequences. With regard to this aspect, it has been already demonstrated that HCWs believing in vaccinations would help control the impact of pandemic on their well-being, since immunized workers probably felt able to resume social activities [14]. Finally, the questionnaire was aimed only at exploring socio-demographic and behavioral aspects and did not include questions about psychological or physical health status of participants. Considering that these may have consequences on the individuals’ behaviors, this represents an important flaw of the study. Despite these potential biases, the use of a large sample of respondent PHWs from different Italian institutions strengthen the usefulness of our findings.

Integrative and multidisciplinary approaches are required to improve health-related behaviors in PHWs. Increasing health literacy in HCWs could help to achieve this goal [22], but also urban design may contribute to health-well-being, creating new models of health promotion [41]. This would further contribute to reducing the negative lifestyle consequences of the COVID-19 pandemic.

## 5. Conclusions

The present findings identified several differences between the lifestyles of Italian PHWs and those of the general Italian general population. Current PHW behavior may be a result of changes during the COVID-19 pandemic in work activity, as the workload of PHWs has greatly increased to cope with the COVID-19 waves in Italy. Further studies are needed to explore in depth PHW behaviors and to identify appropriate health promotion measures to support the adoption of healthy lifestyles in this population during global health emergencies.

## Figures and Tables

**Table 1:** Characteristics of participants (*n* = 1000).

Variable	Participants
Gender *n (*%*)*	
men	365 (36.5)
women	635 (63.5)
Age (years) mean ± SD (range)	40 ± 13.1 (23–79)
Work institution *n (*%*)*	
research	581 (58.1)
healthcare	419 (41.9)
Living conditions	
*n* (%)	
alone	202 (20.2)
with parents	198 (19.8)
with friends/colleagues	104 (10.4)
with partner	210 (21)
with parents and underage children	169 (16.9)
with parents and adult children	117 (11.7)

SD, standard deviation.

**Table 2:** Current health-related conditions and behaviors for the total sample and grouped by gender.

Lifestyle Variable	Whole Sample *n* = 1000 *n* (%)	Men *n* = 365	Women *n* = 635	*p*-Value
BMI category				<0.001
underweight	64 (6.4)	1 (0.3)	63 (9.9)	
normal weight	665 (66.5)	214 (58.6)	451 (71)	
overweight	216 (21.6)	126 (34.5)	90 (14.2)	
obese	55 (5.5)	24 (6.6)	31 (4.9)	
Smoking habit				0.027
no	819 (81.9)	286 (78.4)	533 (84)	
yes	181 (18.1)	79 (21.6)	102 (16)	
Weekly days of sweet food consumption				0.955
0	27 (2.7)	10 (2.7)	17 (2.7)	
1	97 (9.7)	37 (10.1)	60 (9.4)	
2	158 (15.8)	55 (15.1)	102 (16.1)	
3	145 (14.5)	48 (13.2)	97 (15.3)	
4	97 (9.7)	33 (9)	64 (10.1)	
5	112 (11.2)	43 (11.8)	69 (10.9)	
6	64 (6.4)	23 (6.3)	41 (6.4)	
7	300 (30)	116 (31.8)	185 (29.1)	
Physical activity engagement				0.001
no	346 (34.6)	105 (28.8)	241 (38)	
walking/cycling for commuting	215 (21.5)	97 (26.6)	118 (18.6)	
walking/cycling for leisure	60 (6)	28 (7.7)	32 (5)	
exercise/sport outdoors	87 (8.7)	38 (10.4)	49 (7.7)	
exercise/sport in indoor facilities	220 (22)	78 (21.4)	142 (22.4)	
exercise/sport at home	72 (7.2)	19 (5.2)	53 (8.3)	
Time spent watching TV hours/day				0.111
		
<1	407 (40.7)	143 (39.2)	264 (41.6)	
1	258 (25.8)	99 (27.1)	159 (25)	
2	208 (20.8)	78 (21.4)	130 (20.5)	
3	89 (8.9)	25 (6.8)	64 (10.1)	
4	22 (2.2)	10 (2.7)	12 (1.9)	
≥5	16 (1.6)	10 (2.7)	6 (0.9)	
Time spent using smartphone hours/day				0.734
<1	61 (6.1)	26 (7.1)	35 (5.5)	
1	203 (20.3)	77 (21.1)	126 (19.8)	
2	233 (23.3)	87 (23.8)	146 (23)	
3	215 (21.5)	76 (20.8)	139 (21.9)	
4	139 (13.9)	44 (12.1)	95 (15)	
≥5	149 (14.9)	55 (15.1)	94 (14.8)	
Time spent using tablet/PC hours/day				0.004
<1	63 (6.3)	22 (6)	41 (6.5)	
1	90 (9)	42 (11.6)	48 (7.5)	
2	128 (12.8)	50 (13.7)	78 (12.3)	
3	124 (12.4)	49 (13.4)	75 (11.8)	
4	120 (12)	56 (15.3)	64 (10.1)	
≥5	475 (47.5)	146 (40)	329 (51.8)	
Total screen time hours/day				0.078
<1	3 (0.3)	2 (0.5)	1 (0.2)	
1	7 (0.7)	0 (0)	7 (1.1)	
2	33 (3.3)	14 (3.8)	19 (3)	
3	53 (5.3)	25 (6.8)	28 (4.4)	
4	73 (7.3)	28 (7.7)	45 (7.1)	
≥5	831 (83.1)	296 (81.1)	535 (84.2)	
Sleep time hours/night				0.073
≤5	113 (11.3)	41 (11.2)	72 (11.3)	
6	288 (28.8)	117 (32.1)	171 (26.9)	
7	351 (35.1)	118 (32.3)	233 (36.7)	
8	221 (22.1)	81 (22.2)	140 (22.1)	
≥9	27 (2.7)	8 (2.2)	19 (3)	
Doses of COVID-19 vaccine				0.005
0	5 (0.5)	0 (0)	5 (0.8)	
1	23 (2.3)	9 (2.5)	14 (2.2)	
2	715 (71.5)	241 (66)	474 (74.6)	
3	257 (25.7)	115 (31.5)	142 (22.4)	

BMI, body mass index.

**Table 3:** Lifestyle changes during the pandemic for the total sample and grouped by gender.

Lifestyle Variable	Whole Sample *n* = 1000 *n* (%)	Men *n* = 365 *n* (%)	Women *n* = 635 *n* (%)	*p*-Value
Work activity				
no change	476 (47.6)	187 (51.2)	289 (45.5)	0.205
more remote	262 (26.2)	91 (24.9)	171 (26.9)	
more onsite	262 (26.2)	87 (23.8)	175 (27.6)	
Smoking habit				0.273
stopped	21 (2.1)	9 (2.5)	12 (1.9)	
decreased	17 (1.7)	8 (2.2)	9 (1.4)	
no change	106 (10.6)	40 (10.9)	65 (10.2)	
started	18 (1.8)	10 (2.7)	8 (1.2)	
increased	40 (4)	21 (5.8)	19 (3)	
Dietary habits				
improved	164 (16.4)	46 (12.6)	118 (18.6)	0.021
no change	592 (59.2)	234 (64.1)	358 (56.4)	
worsened	244 (24.4)	85 (23.3)	159 (25)	
Sweet food consumption				
decreased	115 (11.5)	37 (10.1)	78 (12.3)	0.039
no change	650 (65)	258 (70.7)	392 (61.7)	
started	18 (1.8)	5 (1.4)	13 (2.1)	
increased	217 (21.7)	65 (17.8)	152 (23.9)	
Body weight				0.216
decreased	201 (20.1)	65 (17.8)	136 (21.4)	
no change	502 (50.2)	180 (49.3)	322 (50.7)	
increased	297 (29.7)	120 (32.9)	177 (27.9)	
Physical activity				0.413
started	16 (1.6)	5 (1.4)	11 (1.7)	
increased	205 (20.5)	65 (17.8)	140 (22)	
no change	390 (39)	147 (40.3)	243 (38.3)	
decreased	389 (38.9)	148 (40.5)	241 (38)	
Time spent watching TV				0.013
decreased	245 (24.5)	72 (19.7)	173 (27.2)	
no change	636 (63.6)	257 (70.4)	379 (59.7)	
increased	119 (11.9)	36 (9.9)	83 (13.1)	
Time spent using smartphone				0.008
decreased	45 (4.5)	9 (2.5)	36 (5.7)	
no change	503 (50.3)	204 (55.9)	298 (46.9)	
increased	452 (45.2)	152 (41.6)	301 (47.4)	
Time spent using tablet/PC				0.215
decreased	41 (4.1)	21 (5.8)	20 (3.2)	
no change	393 (39.3)	144 (22.6)	249 (39.2)	
increased	566 (56.6)	200 (54.8)	366 (57.6)	
Sleep time				0.029
increased	59 (5.9)	13 (3.6)	46 (7.3)	
no change	614 (61.4)	238 (65.2)	376 (59.2)	
decreased	327 (32.7)	114 (31.2)	213 (33.5)	

**Table 4:** Results of the multivariate regression analysis of lifestyle worsening during the pandemic.

Independent Variables	Smoking	Diet	Sweet Food Consumption	Weight	PA	Watching TV	Smartphone Use	Tablet/PC Use	Sleep
	Odds Ratios (95% Confidence Intervals)								
Gender									
women	1.261 (0.845–1.882)	1.038 (0.757–1.422)	1.456 (1.059–2.026) *	0.827 (0.620–1.103)	0.860 (0.655–1.129)	1.399 (0.908–2.155)	1.126 (0.861–1.473)	1 (0.760–1.315)	1.102 (0.828–1.467)
men	Reference	Reference	Reference	Reference	Reference	Reference	Reference	Reference	Reference
Age									
<35 years	0.792 (0.476–1.319)	1.874 (1.261–2.784) *	1.670 (1.126–2.478) *	2.004 (1.381–2.909) **	1.590 (1.118–2.263) *	1.213 (0.711–2.071)	1.251 (0.890–1.759)	1.332 (0.939–1.890)	1.143 (0.798–1.638)
≥35 years	Reference	Reference	Reference	Reference	Reference	Reference	Reference	Reference	Reference
Work institution									
research	0.945 (0.589–1.517)	0.587 (0.409–0.843) *	0.608 (0.424–871) *	0.711 (0.509–0.992) *	0.550 (0.402–0.752) **	0.790 (0.500–1.251)	1.020 (0.752–1.382)	0.765 (0.619–1.154)	0.681 (0.494–0.939) *
healthcare	Reference	Reference	Reference	Reference	Reference	Reference	Reference	Reference	Reference
Living conditions									
with parents	1.022 (0.564–1.852)	1.004 (0.629–1.602)	1.001 (0.631–1.588)	1.236 (0.798–1.914)	1.035 (0.673–1.590)	0.787 (0.413–1.501)	1.158 (0.765–1.751)	0.730 (0.477–1.118)	0.932 (0.601–1.446)
with friends/colleagues	0.623 (0.322–1.206)	0.780 (0.433–1.404)	0.577 (0.316–1.056)	0.428 (0.232–0.790) *	1.664 (1.002–2.761) *	0.673 (0.309–1.466)	1.545 (0.934–2.555)	1.074 (0.640–1.801)	0.693 (0.399–1.205)
with partner	1.010 (0.570–1.789)	0.993 (0.636–1.553)	0.788 (0.502–1.237)	1.309 (0.863–1.985)	1.110 (0.740–1.665)	0.931 (0.505–1.716)	0.710 (0.476–1.057)	0.865 (0.579–1.291)	0.844 (0.556–1.283)
with partner and underage children	3.023 (1.299–7.034) *	0.919 (0.552–1.528)	0.823 (0.497–1.366)	0.856 (0.521–1.406)	1.422 (0.909–2.223)	0.532 (0.259–1.093)	1.121 (0.726–1.730)	1.684 (1.065–2.663) *	1.039 (0.660–1.635)
with partner and adult children	1.777 (0.771–4.096)	0.326 (0.159–0.667) *	0.364 (0.183–0.726) *	1.032 (0.594–1.792)	0.836 (0.497–1.407)	1.021 (0.486–2.144)	0.721 (0.437–1.190)	0.872 (0.528–1.441)	0.547 (0.317–0.942) *
alone	Reference	Reference	Reference	Reference	Reference	Reference	Reference	Reference	Reference
Work activity									
more remote	0.617 (0.382–0.996) *	1.982 (1.385–2.838) **	1.265 (0.876–1.827)	0.950 (0.672–1.345)	1.306 (0.945–1.804)	1.264 (0.712–2.241)	1.346 (0.977–1.854)	3.314 (2.358–4.656) **	2.065 (1.482–2.877) **
more onsite	0.718 (0.447–1.152)	0.977 (0.664–1.439)	0.980 (0.670–1.431)	0.959 (0.677–1.357)	1.088 (0.788–1.502)	4.609 (2.883–7.370) **	1.649 (1.205–2.258) **	2.223 (1.614–3.060) **	1.268 (0.903–1.781)
no change	Reference	Reference	Reference	Reference	Reference	Reference	Reference	Reference	Reference

* *p*-Value < 0.05; ** *p*-value < 0.01.

## Data Availability

All data presented are available upon request from the corresponding author (C.N.).
